# Supplementary material for: Investigation of phytochemical and biochemical attributes of hypoglycemic activity of Atriplex crassifolia (C.A. Mey) extracts in alloxan diabetic animal model
Source: PLoS One. 2025 Sep 25;20(9):e0333425. doi: 10.1371/journal.pone.0333425 (PMC12463247; doi:10.1371/journal.pone.0333425)
Supplement: S2 File — (DOCX) [file pone.0333425.s002.docx]

**Supplementary file of Manuscript “****Investigation of phytochemical and biochemical attributes of hypoglycemic activity of *Atriplex crassifolia* (C.A. Mey) extracts in alloxan diabetic animal model”**

**S1 Table: Biochemical parameters associated with hyperlipidemias in normal and hyperglycemic rats treated with active extracts of *A. crassifolia***

| **Group** | **Dose**  **(mg/kg)** | **Cholesterol mg/dl**  **<200** | **Triglycerides mg/dl**  **<150** | **HDL**  **mg/dl**  **35-65** | **VLDL**  **mg/dl**  **<25** | **LDL**  **mg/dl**  **<130** |
| --- | --- | --- | --- | --- | --- | --- |
| **Normal control** | Vehicle | 119±  1.01^***^ | 135.5±  2.51^***^ | 23.2±  0.74^***^ | 26.8±  1.02^***^ | 67.7±  0.55^***^ |
| **Diabetic control** | Vehicle | 293.7±  0.75 | 219.3±  0.76 | 37.9±  0.79 | 43.9±  0.15 | 140.8±  0.63 |
| **Standard control** | 10 | 124.7±  0.24^***^ | 121.1±  0.93^***^ | 41.4±  0.65^*^ | 22.6±  0.19^***^ | 117.9±  2.51^***^ |
| **ACEA** | 250 | 205.1±  0.99^***^ | 216.75±  5.24ns | 35.60±  0.40 | 42.9±  1.41ns | 119.3±  1.14^***^ |
|  | 500 | 201.2±  0.75^***^ | 155.1±  0.91^***^ | 47.1±  0.91^***^ | 20.4±  0.63^***^ | 121.5±  0.53^***^ |
|  | 1000 | 120.0±  1.06^***^ | 132.5±  0.25^***^ | 55.6±  0.40^***^ | 21.9±  0.15^***^ | 120.1±  0.91^***^ |
| **ACNB** | 250 | 137.1±  0.96^***^ | 127.25±  1.23^***^ | 35.6±  0.41^ns^ | 31.4±  0.61^**^ | 124.1±  0.93^**^ |
|  | 500 | 185.1±  0.92^***^ | 124.10±  0.96^***^ | 52.1±  0.97^***^ | 34.0±  0.83^**^ | 141.8±  0.62^ns^ |
|  | 1000 | 203.1±  1.97^***^ | 182.6±  0.45^***^ | 62.1±  0.15^***^ | 36.2±  0.45^**^ | 127.8±  1.06^**^ |

Results are shown as mean ± SEM, ACEA: *A. crassifolia* Ethyl acetate extract, ACNB: *A. crassifolia* n-butanol extract, *=<0.01, **=<0.001, ***=<0.0001 respectively by one way ANOVA followed by Dunnet’s multiple comparison test (n=5)

**S2 Table: Biochemical assessment of liver and renal function in normal and hyperglycemic rats treated with active extracts of *A. crassifolia***

| **Group** | **Dose**  **(mg/kg)** | **Urea**  **(mg/dl)**  **10-50** | **Creatinine**  **(mg/dl)**  **0.60-1.30** | **Bilirubin**  **(mg/dl)**  **0.30-1.20** | **AST**  **(mg/dl)**  **10-40** | **ALT**  **(mg/dl)**  **10-45** | **ALP**  **(mg/dl)**  **98-279** |
| --- | --- | --- | --- | --- | --- | --- | --- |
| **Normal control** | Vehicle | 45.90±  0.30^***^ | 0.46±  0.01^***^ | 0.42±  0.01^***^ | 35.75±  0.25^***^ | 42.40±  0.40^***^ | 201.65±  0.65^***^ |
| **Diabetic control** | Vehicle | 79.15±  0.55 | 0.78±  0.05 | 1.27±  0.01 | 101.95±  0.05 | 112.60±  0.40 | 323.20±  1.80 |
| **Standard control** | 10 | 46.05±  0.15^***^ | 0.37±  0.01^***^ | 0.42±  0.01^***^ | 44.15±  0.15^***^ | 45.95±  0.05^***^ | 212.75±  0.75^***^ |
| **ACEA** | 250 | 64.05±  0.55^***^ | 0.64±  0.01^**^ | 1.06±  0.05^***^ | 88.25±  0.75^**^ | 98.45±  0.45^***^ | 303.65±  1.35^*^ |
|  | 500 | 62.15±  0.35^***^ | 0.49±  0.01^***^ | 1.02±  0.01^***^ | 74.60±  0.60^***^ | 84.85±  0.15^***^ | 283.60±  0.40^***^ |
|  | 1000 | 48.25±  0.35^***^ | 0.48±  0.01^***^ | 0.74±  0.01^***^ | 52.60±  0.60^***^ | 62.65±  0.65^***^ | 261.75±  0.25^***^ |
| **ACNB** | 250 | 52.25±  0.45^***^ | 0.63±  0.01^**^ | 0.84±  0.01^***^ | 55.95±  0.05^***^ | 60.10±  1.10^***^ | 286.75±  2.25^**^ |
|  | 500 | 49.60±  0.10^***^ | 0.60±  0.01^***^ | 0.53±  0.02^***^ | 49.60±  0.60^***^ | 51.90±  0.10^***^ | 236.30±  1.30^***^ |
|  | 1000 | 45.55±  0.15^***^ | 0.37±  0.02^***^ | 0.42±  0.05^***^ | 44.65±  0.35^***^ | 46.65±  0.35^***^ | 217.10±  1.90^***^ |

Results are shown as mean ± SEM, ACEA: *A. crassifolia* Ethyl acetate extract, ACNB: *A. crassifolia* n-butanol extract, *=<0.01, **=<0.001, ***=<0.0001 respectively by one way ANOVA followed by Dunnet’s multiple comparison test

**Sup Table 3: Oral glucose tolerance test of ACNB of *A. crassifolia* in alloxan induced diabetic rats (individual readings)**

|  | **ACNB_500_120 min** | **ACNB_1000_-30 min** | **ACNB_1000_**  **Baseline** | **ACNB_1000_30 min** | **ACNB_1000_60 min** | **ACNB_1000_120 min** |
| --- | --- | --- | --- | --- | --- | --- |
| Rat_1 | 142 | 268.9 | 257.4 | 250.3 | 121.85 | 114.25 |
| Rat_2 | 143.9 | 267.4 | 255.5 | 251.5 | 121.96 | 112.17 |
| Rat_3 | 143.2 | 265.4 | 253.5 | 248.4 | 122.66 | 111.03 |
| Rat_4 | 143.3 | 264.5 | 255.4 | 251.0 | 122.63 | 112.25 |
| Rat_5 | 143.5 | 265.9 | 257.9 | 250.3 | 119.45 | 111.25 |
| Mean | 143.2 | 266.4 | 255.9 | 250.3 | 121.71 | 112.19 |
| S.D | 0.7 | 1.7 | 1.8 | 1.2 | 1.3 | 1.3 |
| S.E | 0.3 | 0.8 | 0.8 | 0.5 | 0.6 | 0.6 |
