# Supplementary material for: Investigation of phytochemical and biochemical attributes of hypoglycemic activity of Atriplex crassifolia (C.A. Mey) extracts in alloxan diabetic animal model
Source: PLoS One. 2025 Sep 25;20(9):e0333425. doi: 10.1371/journal.pone.0333425 (PMC12463247; doi:10.1371/journal.pone.0333425)
Supplement: Attachment — Submitted filename: Response sheet A. crassifolia Plos.docx [file pone.0333425.s004.docx]

**Response Sheet**

**[PONE-D-24-40010] - [EMID:0053111b80385d9c]**

We appreciate you giving us the chance to make revisions to our manuscript, " Investigation of phytochemical and biochemical attributes of hypoglycemic activity of *Atriplex crassifolia* (C.A. Mey) extracts in alloxan diabetic animal model" Your insightful and beneficial comments have helped us to improve our manuscript. Every comment has been thoroughly reviewed, and appropriate modifications have been made. We trust that all the modifications have been carried out correctly, and we hope to receive approval. All the corrections and responses to reviewer comments are as follows.

| **Comments** | **Response** |
| --- | --- |
| **Journal Requirements** |  |
| Please ensure that your manuscript meets PLOS ONE's style requirements, including those for file naming. The PLOS ONE style templates can be found at | We have corrected the manuscript accordingly |
| To comply with PLOS ONE submissions requirements, in your Methods section, please provide additional information regarding the experiments involving animals and ensure you have included details on (1) methods of sacrifice, (2) methods of anesthesia and/or analgesia, and (3) efforts to alleviate suffering. | The details have been provided in the manuscript and the supplementary file. |
| We note that your Data Availability Statement is currently as follows: [All relevant data are within the manuscript and its Supporting Information files.]  For example, authors should submit the following data:  - The values behind the means, standard deviations and other measures reported;  - The values used to build graphs;  - The points extracted from images for analysis. | We have included all the data generated in the manuscript and supplementary file. |
| **Editor Comments** |  |
| The manuscript entitled “Investigation of phytochemical and biochemical attributes of hypoglycemic activity of *Atriplex crassifolia* (C.A. Mey) extracts in alloxan diabetic animal model” is interesting although poorly written and presentation is very poor | Thank you for your comment. We have tried to improve the writing and presentation of manuscript |
| The antioxidant activity of extract was evaluated with highest dose 1000 µg/ml; how the IC_50_ values of n-hexane extract, and dichloromethane extract of *A. crassifolia* was evaluated, even higher than the highest concentration of experimentation? | Thank you for your comment. IC_50_ value represents half of the maximal inhibitory concentrations against free radicals. n-hexane and dichloromethane displayed higher IC_50_ values even at the highest concentration of 1000 µg/ml so they could not reach the 50% inhibition that shows they have less antioxidant potential. |
| **The figures in the manuscript are very poor quality, and need improvement. Please improve the manuscript in due time.** | **We have tried to improve figures.** |
| **Reviewer 1** |  |
| Figures 1-7 are often illegible. Authors need to make improvements so that the legend, axis etc are clearly readable. | Thank you for your suggestion. We have tried to improve the figures as suggested. |
| Authors should comment on lack of dose-dependent correlation for several extracts in free radical scavenging assay in Table 2. | Thank you for your comment. We acknowledge the lack of dose dependent correlation with various extracts in DPPH free radical scavenging assay. This variability in antioxidant potential may be attributed to the difference in the composition of bioactive compounds in the tested extracts. The non-linear relationship might be due to effectiveness of certain phytoconstituents at specific concentrations. Scavenging activity of certain constituents plateaus beyond certain concentration (ceiling effect) that leads to little or no response at particular concentrations (Huang et al., 2005).  Huang, D., Ou, B., & Prior, R. L. (2005). The chemistry behind antioxidant capacity assays. *Journal of Agricultural and Food Chemistry*, 53(6), 1841-1856. <https://doi.org/10.1021/jf030723c> |
| Authors should comment on the relationship/evidence behind potential presence of antioxidants and relevance as therapeutic agents. | Thank you for your suggestion. We have added the therapeutic relevance of antioxidants in Lines 325-331 as follows  The therapeutic activity of most of the plants is attributed to their potent antioxidant capability. Free radicals play a vital role in pathogenesis of various diseases. Phytoconstituents particularly phenols and flavonoids serve as the best exogenous antioxidants. They not only prohibit ROS, but also boost the endogenous defense mechanism of the body against free radicals. Phenols and flavonoid containing plants exhibit a wider range of biological activities than the conventional hydrogen donating agents. In our study, the antioxidant capabilities of *A.crassifolia* may be responsible for its significant antidiabetic effects. |
| Authors should comment on variable response to ACEA and ACNB extracts at different doses, between 500 and 1000. | Thank you for your valuable suggestion.  The non-linear dose-response relationship of the extracts might be due to altered therapeutic behavior of the phytoconstituents. At higher doses, receptor saturation or downstream signaling may result in non-proportional therapeutic efficacy. Receptor binding limitations might lead to a plateau in glucose lowering capability. Moreover, the bioavailability of the bioactive compounds might decrease at higher concentrations due to enhanced metabolism. The plant constituents exhibit both synergistic and antagonistic properties at various concentrations. If the antagonistic behavior predominates at higher concentrations, this may lead to a variable dose-response relationship.  Efferth T, Koch E. Complex interactions between phytochemicals. The multi-target therapeutic concept of phytotherapy. Current drug targets. 2011 Jan 1;12(1):122-32. |
| Statement "Hence, plants with high antioxidant capacity have a notably beneficial impact on human well-being and physical condition." on line 320 is not justified. | Thank you for your comment. We have justified the statement as follows  Hence, plants with high antioxidant capacity have a notably beneficial impact on human well-being and physical condition. This is due to their enhanced therapeutic efficacy due to the presence of antioxidant phytoconstituents as discussed above. |
| Results in Table 6 are somewhat surprising and contradictory to results presented earlier. ACEA extracts did not demonstrate any efficacy at dose lower than 1000 mg, however, there seems to be a correlation to weight gain even at dose as low as 250 mg. On the other hand, ACNB extract were shown to be effective at even lower doses on the other experiments, but only the 1000 mg really leads to weight gain. Could authors please comment on this? | Thank you for your valuable comment. ACEA did not show hypoglycemic effect at dose lower than 1000 mg, however, it displayed a weight gain at dose as low as 250 mg. It might be proposed that the mechanisms of weight modulation and antidiabetic activity might not be aligned, and the weight gain was probably due to some other underlying mechanism. ACEA might contain compounds that cause wight gain independently through some metabolic effects that are not linked with glucose regulation. Conversely, ACNB displayed hypoglycemic activity at the lowest dose of 250 mg but caused weight gain at 1000 mg. it might contain constituents that cause hypoglycemic affect primarily without affecting weight and at high doses, additional metabolic effects might cause weight gain. This needs to be further evaluated through studies focused on appetite regulation, lipid metabolism and insulin sensitivity to figure out the exact mechanism behind weight changes. |
| Reviewer 2 |  |
| Its a comprehensive study, which is appreciable. | We appreciate your valuable comment |
| The study does not provide detailed information on the specific molecular mechanisms by which the *Atriplex crassifolia* extracts exert their antidiabetic effects. While it mentions the presence of compounds like myricetin, quercetin, and sinapic acid, which are known to have antidiabetic properties, it doesn't elucidate how these compounds specifically act in this context. The research fails to identify or investigate specific drug targets. There's no mention of receptor binding studies, or other experiments that would pinpoint the exact molecular targets of the active compounds in the extracts. Moreover, this study doesn't explore the potential interactions between the various compounds found in the extracts, nor does it investigate how these compounds might interact with known antidiabetic drug targets or pathways. These are indeed significant limitations of the study. | Thank you for your valuable comment. We have included the limitations in Lines 419- 425.  The hypoglycemic mechanism of myricetin, quercetin and sinapic acid have been explained between lines (264-275). We appreciate the insightful comments of the reviewer regarding the drug targets and the exact molecular mechanisms underlying the hypoglycemic potential of *Atriplex crassifolia* extracts. The primary focus of the current study was to explore the hypoglycemic effect of *Atriplex crassifolia* extracts and the possible bioactive constituents responsible for it. Quercetin, myricetin and sinapic acid are well known antidiabetic compounds and they exert hypoglycemic effect through various mechanisms as stated in text (lines 264-275). However, further studies are required to understand the exact molecular targets as well as pathway analysis for a more comprehensive study. We also acknowledge another limitation of the study regarding the potential interactions between various compounds found in extracts and the synergistic effects of active constituents of *Atriplex crassifolia* with other antidiabetic agents. Our study primarily serves as the initial step in phytochemical constituents profiling and establishing their therapeutic relevance with the aim of contributing some valuable preliminary data and supporting the need to further explore the plant constituents. We have added the limitations of our study in the discussion section and proposed the future recommendations for advanced studies on *Atriplex crassifolia.* |
