# Supplementary material for: Investigation of phytochemical and biochemical attributes of hypoglycemic activity of Atriplex crassifolia (C.A. Mey) extracts in alloxan diabetic animal model
Source: PLoS One. 2025 Sep 25;20(9):e0333425. doi: 10.1371/journal.pone.0333425 (PMC12463247; doi:10.1371/journal.pone.0333425)
Supplement: Attachment — Submitted filename: Response Sheet Sep 2025.docx [file pone.0333425.s005.docx]

Response Sheet **[PONE-D-24-40010R1] - [EMID:d0a3c1757778940b], August 2025**

Investigation of phytochemical and biochemical attributes of hypoglycemic activity of Atriplex crassifolia (C.A. Mey) extracts in alloxan diabetic animal model

| Comment | Response |
| --- | --- |
| Journal Requirements:  If the reviewer comments include a recommendation to cite specific previously published works, please review and evaluate these publications to determine whether they are relevant and should be cited. There is no requirement to cite these works unless the editor has indicated otherwise.   Please review your reference list to ensure that it is complete and correct. If you have cited papers that have been retracted, please include the rationale for doing so in the manuscript text, or remove these references and replace them with relevant current references. Any changes to the reference list should be mentioned in the rebuttal letter that accompanies your revised manuscript. If you need to cite a retracted article, indicate the article’s retracted status in the References list and also include a citation and full reference for the retraction notice. | We have carefully reviewed the references. One of the references at No. 75 had been withdrawn.  Siddique NA, Mujeeb M, Najmi AK, Akram M. Evaluation of antioxidant activity, quantitative estimation of phenols and flavonoids in different parts of Aegle marmelos. Afr J Plant Sci. 2010 Jan 31;4:1: 1-5.(Withdrawn).  We have removed the withdrawn reference and replaced the relevant text and citation with the other one. The reference has been highlighted in red in the reference list. |
| Reviewer #3:  1. The figures are still illegible. The authors need to make improvements to make them clear and readable. Mention the improvements made with the figures to meet the standards of the journal. | Thank you for your valuable comment. We have attempted to enhance the figures by increasing their dpi to comply with the journal's specifications. |
| 2. Do the authors consider alternative analyses for dose-dependent responses? | We thank the reviewer for the valuable suggestion. Our current study design aimed at evaluating the hypoglycemic activity of *A.crassifolia* extracts at various doses. As per your comment, yes, dose-dependent analysis would provide additional insights, Future studies might be carried out to observe a potential dose-related effect, which we have now acknowledged and discussed in the revised manuscript under the “Conclusion” section. |
| 3. The explanation regarding the compounds, although acceptable, still seems vague. Elaborate or use a graphical representation | I appreciate your insightful remark. We have attempted to elucidate the function of compounds and rephrased certain sentences to enhance clarity. We expanded the text but did not include any graphical representations, as the manuscript already contains eight figures. |
| 4. The response to the comment on the statement on antioxidants improving well-being is generic. Provide clinical evidence | We are thankful to reviewer for the valuable suggestion. We have provided clinical evidence of antioxidants improving well-being with reference. We have added the following statement  A crucial aspect of general well-being is the improvement of metabolic health. Meta-analysis of randomized controlled trials has substantiated clinical evidence that antioxidant supplementation in obese persons significantly lowers fasting blood glucose levels, enhances insulin resistance, and diminishes lipid peroxidation. |
| There are issues with the language used. Use concise scientific language. | Thank you for your valuable comment. We have tried to review the manuscript and rectify the scientific language where needed. |
